# Studying forced expiratory volume at 1 second over menstrual segments in asthmatic and non-asthmatic women: assessing protocol feasibility

**DOI:** 10.1186/1756-0500-5-261

**Published:** 2012-07-06

**Authors:** Ganesa Wegienka, Ewa Hasiec, Homer Boushey, Christine Cole Johnson, Ronald Strickler, Edward Zoratti, Suzanne Havstad

**Affiliations:** 1 Department of Public Health Sciences, Henry Ford Hospital, Detroit, MI, USA; 2 Division of Allergy and Immunology, University of California, San Francisco, USA; 3 Department of Women’s Health, Henry Ford Hospital, Detroit, MI, USA; 4 Division of Allergy and Clinical Immunology, Henry Ford Hospital, Detroit, MI, USA; 5 Department of Public Health Sciences, Henry Ford Hospital, 1 Ford Place, 3E, Detroit, MI 48202, USA

**Keywords:** Sex hormones, Lung function, Estrogen, Progesterone, Menstrual segment

## Abstract

**Background:**

Sex hormones may play an important role in observed gender differences in asthma incidence and severity, as well as in the observed changes in asthma symptoms during times of hormonal fluctuation (i.e.; premenstrual, pregnancy, etc.). This pilot study sought to demonstrate the feasibility of data collection methods to investigate the effects of sex hormones on lung function in women.

**Findings:**

A cohort of 13 women (6 with and 7 without prior asthma diagnoses) who were having menstrual periods and were not taking hormones collected urine samples daily for measurement of estrogen (estrone E1C) and progesterone (Pregnanediol-glucuronide PDG) metabolites over the course of a menstrual segment (bleeding episode plus the following bleeding-free interval). Hormones were used to estimate menstrual segment phase (follicular versus luteal) based on a published algorithm. Daily bleeding and FEV1 measurements were recorded and percent predicted FEV1 was calculated. Percent predicted FEV1 decreased over the course of the follicular but not the luteal phase. More specifically, among women without a prior asthma diagnosis, the E1C/PDG ratio and E1C and PDG were individually associated with FEV1 in the follicular phase. No associations were found between hormones and percent predicted FEV1 in the luteal phase or among asthmatic women. E1C was associated with FEV1 in the five days before bleeding onset only among non-asthmatic women.

**Discussion:**

A study of contiguous daily hormones and symptoms over menstrual segments from a large group of women with and without asthma is needed to better determine within-woman cyclicity of the observed patterns.

## Findings

### Background

Compared with men, women are more severely affected by asthma including a greater prevalence (8.8% versus 6.4%), more frequent emergency department visits for asthma (age-adjusted: 65 visits/10,000 persons versus 62 visits/10,000 persons) and higher hospital admission rates for asthma (age-adjusted: 19/10,000 persons versus 14/10,000 persons) [1]. Limited reports in the literature suggest that asthma symptoms worsen during times of hormonal change such as puberty, pregnancy, menopause and the premenstrual stage [2]. Based on these findings, it is reasonable to expect that sex hormones may play a role in asthma development and exacerbation; however, any underlying mechanisms between hormones and lung function in women have not been clearly defined [3,4]. Most prior investigations of the role of sex hormones in lung function and asthma severity in women have been plagued by poor study design, limited data collection and select study populations.

Our overall research questions were: what are the associations between sex hormones and lung function in women and do any observed associations differ by asthma status?

To determine the effects of sex hormones on lung function (forced expiratory volume at 1 second (FEV1)), we had five main scientific objectives in our study population of asthmatic and non-asthmatic women. The work presented here was a pilot study to test the feasibility of methods for addressing our proposed research objectives. The objectives were to determine whether:

1) FEV1 varies over the menstrual segment;

2) Any observed patterns of FEV1 variability are the same before and after the estimated day of ovulation;

3) FEV1 varies in the 5 days before the onset of bleeding (so-called premenstrual worsening);

4) Changes in absolute hormone levels (estrogen and progesterone metabolites) and/or the ratios of hormones are associated with FEV1 changes; and

5) Any observed associations differ between asthmatic and non-asthmatic women.

We investigated these objectives and tested our data collection methods for our pilot study comprised of a cohort of 13 women who completed FEV1 measurements with a commercially available meter at home daily over a 6 week period to cover the entire course of one menstrual segment (bleeding episode plus bleeding-free interval). The participants also collected urine samples daily for measurement of estrogen (estrone E1C) and progesterone (Pregnanediol-glucuronide PDG) metabolites. We detail the methods used and the lessons learned that can be employed in future studies of the association between sex hormones and lung function.

## Methods

### Study population

Women were recruited from employees of Henry Ford Health System, Detroit, Michigan. Women having menstrual periods and not taking any hormones were recruited. There were no age restrictions. Women provided written informed consent. This research was approved by the HFHS Institutional Review Board (#5076). Using a screening interview, we recruited a population enriched with asthmatic women in order to allow comparisons between asthmatic (prior doctor diagnosis of asthma) and non-asthmatic women.

### Study protocol

Protocol duration was six weeks and women were asked to begin the protocol a few days prior to when they expected to begin their next menstrual period. Women completed a baseline interview about their basic and reproductive health. Women were also asked whether they had ever been diagnosed with asthma by a doctor and to report all medication they were taking for any reason. We did not ask if they usually had worsening of their asthma symptoms at any particular time during their menstrual cycle. Women were instructed to complete a daily diary about bleeding and medication use. The first day of the bleeding episode was taken from the daily diary.

Participants were asked to collect a sample of their first morning urine every day of the study. Women were given a pink rubber duck toy to keep in their bathroom to remind them to collect their urine. Samples were frozen immediately until analyzed for urinary metabolites of estrogen (E1C) and progesterone (PDG). The participants were also asked to measure FEV1 every morning (as soon as possible after waking and before any medication use) using a Microlife Digital Peak Flow/FEV1 Meter (Microlife USA, Inc.; Clearwater, FL, USA). Women were instructed on how to use the meter by the same research staff member and were told not to add additional measurements if a measurement was missed. The meters are electronic and each measurement is stamped with the date and time, thus preventing women from completing measurements for missed events. Percent predicted FEV1 was calculated to adjust for age, height, gender and race using the NHANESIII data for the reference values [5]. FEV1 reference values do not exist for menstrual phase. For this research, we used the terminology recommended by the WHO and defined a menstrual segment as a bleeding episode followed by a bleeding-free interval [6]. When the women completed the protocol, they were asked to complete a questionnaire asking them to list comments about things that were easy to complete, things that were difficult to complete and what she would change about the study to make it easier to complete the protocol. Comments were open-ended.

### Hormone measurement

Numerous epidemiologic studies have measured daily hormones in other disease areas, namely to study fertility or ovarian function [7,8]. The methods, which utilize first daily urine samples collected by the women and placed in their home freezer, are well established in the field of reproductive epidemiology and have been proven successful in women of all ages and of various ethnic and socioeconomic backgrounds. Urine samples were processed at the CLASS laboratory at the University of Michigan using methods described below. We used the algorithm of Baird et al. to establish an estimated ovulation day based on daily E1C and PDG measurements and their ratios and rates of change. The algorithm was developed after 25-30% of sample cycles from a cohort study with daily urine samples failed to demonstrate LH peaks. These methods have been published in detail and have been previously adapted for research in fertility; however, they do not appear to have been applied to respiratory research [9]. Although ovulation is estimated, we refer to the period prior to the estimated date of ovulation as the follicular phase and the period after as the luteal phase. Hormone metabolites were adjusted for creatinine levels (ratio of metabolite to creatinine).

### Urinary estrone conjugate and urinary pregnanediol-glucuronide assays

The Estrone-conjugate (E1C) assay and the Pregnanediol-glucuronide (PDG) assay are competitive immunoassays [8,10] with manual steps and an off-line incubation. Details have been published [11].

### Urinary creatinine assay

Creatinine was measured with a spectrophotometric assay. Details have been published [11].

### Statistical methods

To assess within-woman change in FEV1 over a menstrual segment, a time variable was defined in terms of days in relation to estimated ovulation date. For measurements during the follicular phase, days were expressed as negative numbers; for measurements in the luteal phase, the number of days was positive. The time variable was thus anchored to estimated ovulation date and multilevel models (MLM) were fit using full maximum likelihood methods and included both random and fixed effects (called mixed effect models, PROC MIXED (SAS V9.2, Release 2, Cary, NC)). In the model examining the 5 days before the onset of bleeding, days were negative and anchored around the first day of bleeding (day 0). We have previously employed this analytical method [11,12].

Our modeling approach allowed us to directly address our research aims related to phase. Random effects accounted for the correlation within-individual (repeated measures). The multilevel model approach is flexible enough to allow and account for variability in the spacing of collection points and complete data are not needed for inclusion in the model. We employed a two-level MLM in order to address both within-individual change over time and between-individual change as affected by additional covariates. Five of six asthmatic women took asthma medication during the study. Thus, no additional analyses of the effects of asthma medication usage were included as there were not enough women with asthma who were not taking medication to assess separate effects of asthma status and medication.

Additional generalized estimating equation (GEE) models and interaction terms were used to examine the relationships between percent predicted FEV1 and E1C and PDG (alone and adjusted for the other) and the ratio of E1C/PDG within each phase (follicular or luteal) or in the 5 days prior to bleeding onset. The specific levels of the sex hormones in the models were selected from the same day as the percent predicted FEV1. The associations were examined for all women and separately for women with and without a prior asthma diagnosis. Hormone levels were log transformed to meet model assumptions.

## Results

Sixteen women enrolled in the study that occurred September-December 2009. Details of the study population were previously published [11]. Three women were excluded because the hormone levels did not change enough to indicate ovulation based on the algorithm and the date of ovulation could not be estimated. Three women did not have complete menstrual segments and analyses were run with and without these women. Of the 13 women in the analyses, 6 (46.2%) reported a prior asthma diagnosis – 5 (38.5%) took asthma medication during the study. The asthmatic women reported having asthma symptoms or wheezing in the last year. Table 1 presents their age at first asthma attack, any asthma medications they reported using in the last year, report of nasal allergies and age at menarche. The age range of all women was 24–48 years with a mean (SD) age of 36.1 (8.0) years and 9 (69.2%) women had a prior pregnancy (all with at least one live birth).

**Table 1:** Details about the asthmatic women included in the analyses

Participant	Age at First Asthma Attack	Type of Medication Used	Self Reported Nasal Allergies?	Age at menarche
A	12	Advair, Albuterol	Yes	12
B	16	Albuterol	No	13
C	26	Advair, Ventilin	Yes	12
D	18	Singulair, Advair, Albulterol	Yes	12
E	35	Albuterol	Yes	13
F	Could not report	None	No	11

The 13 women contributed 309 measurements collected on the first day of the menstrual segment to the last day before the start of the next menstrual segment (number of FEV1 measurements per woman median = 25; range = 14 to 28). Overall, the average percent predicted FEV1 was 93.0% (standard deviation = 11.3%). FEV1 means by phase and asthma status are presented in Table 2.

**Table 2:** Mean percent predicted FEV1 over the menstrual segment stratified by phase and asthma status (n = 13)

	All Women (n = 13)	Non-Asthmatic women (n = 7)	Asthmatic Women (n = 6)
Overall	93.0 (11.3)	92.9 (11.9)	93.2 (10.7)
Follicular phase	95.7 (10.6)	96.5 (11.1)	94.9 (10.0)
Luteal Phase	90.4 (11.4)	89.5 (11.6)	91.3 (11.1)
5 Days prior to onset of bleeding	93.0 (11.4)	92.6 (12.3)	93.5 (10.6)

**Figure 1:**
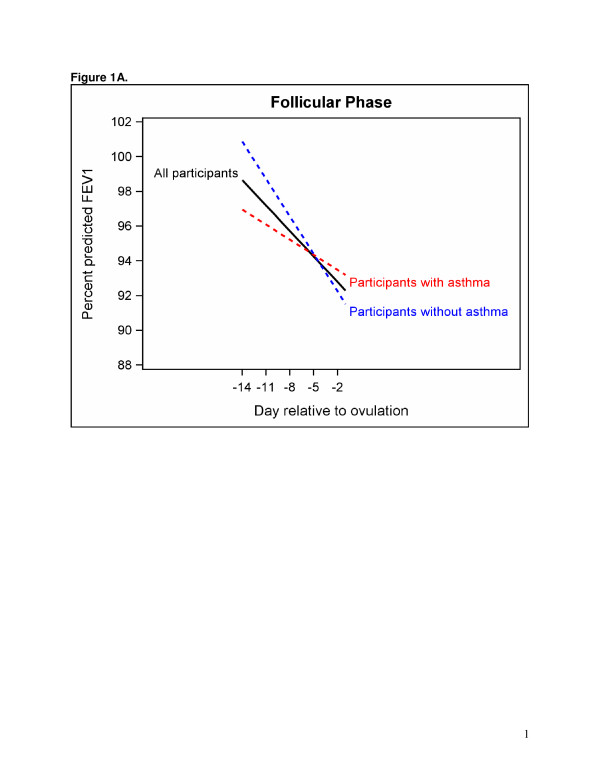
**A plot of the association between day relative to ovulation and percent predicted FEV1 in the follicular phase. A.** The blue dashed line is for participants without asthma and the red dashed line is for participants with asthma. The solid line is the groups combined. **B.** A plot of the association between day relative to ovulation and percent predicted FEV1 in the luteal phase. The blue dashed line is for participants without asthma and the red dashed line is for participants with asthma. The solid line is the groups combined.

A linear model best represented the association between segment day and FEV1 (Table 3). An indicator variable for follicular or luteal phase had a significant interaction (p = 0.003) with menstrual segment day, thus separate models are presented for the follicular (Figure 1a) and luteal phases (Figure 1b). The models indicate that the number of days relative to the estimated date of ovulation is associated with percent predicted FEV1 during the follicular phase. This result indicates an overall decrease in FEV1 as the day of ovulation approaches (p < 0.001). No statistically significant association was found between menstrual segment day and percent predicted FEV1 in the five days prior to the onset of menses or the luteal phase (Table 3); however, the overall pattern suggests FEV1 decreases in asthmatic women and increases in non-asthmatic women in the luteal phase (Figure 1b).

**Table 3:** Models of percent predicted FEV1 over the menstrual segment stratified by phase (n = 13)

	Beta (s.e.)	p-value
Follicular phase		
Intercept	91.8 (2.6)	<0.001
Number of days relative to estimated day of ovulation	−0.49 (0.11)	<0.001
Luteal phase		
Intercept	90.6 (2.9)	<0.001
Number of days relative to estimated day of ovulation	0.02 (0.10)	0.84
5 Days prior to Onset of Menses		
Intercept	92.7 (3.6)	<0.001
Number of days relative to onset of Menses	−0.02 (0.45)	0.97

**Figure 2:**
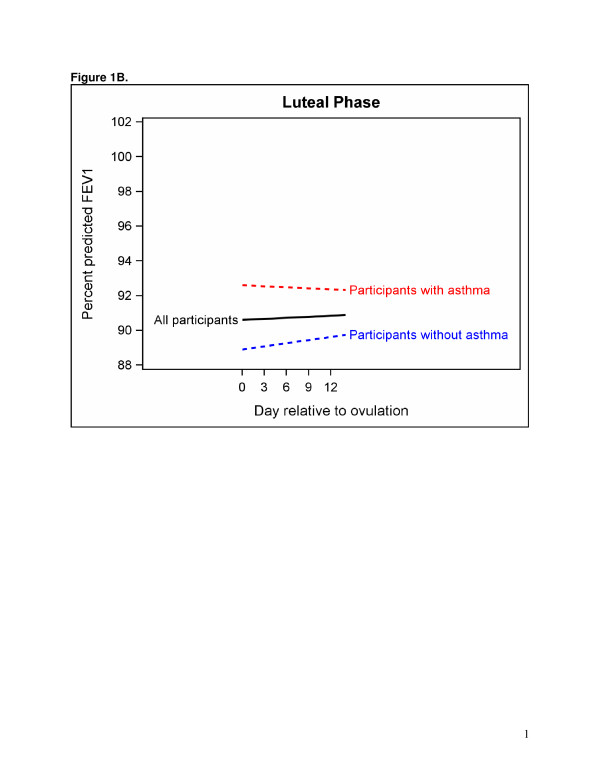
**The log of E1C/PDG ratio of hormone levels (x axis) associated with percent predicted FEV1 (y axis) in the follicular phase among all women.** For purposes of illustrating the relationship between hormones and FEV1, the menstrual segment day is held constant at 7 days prior to ovulation date for the plot.

**Figure 3:**
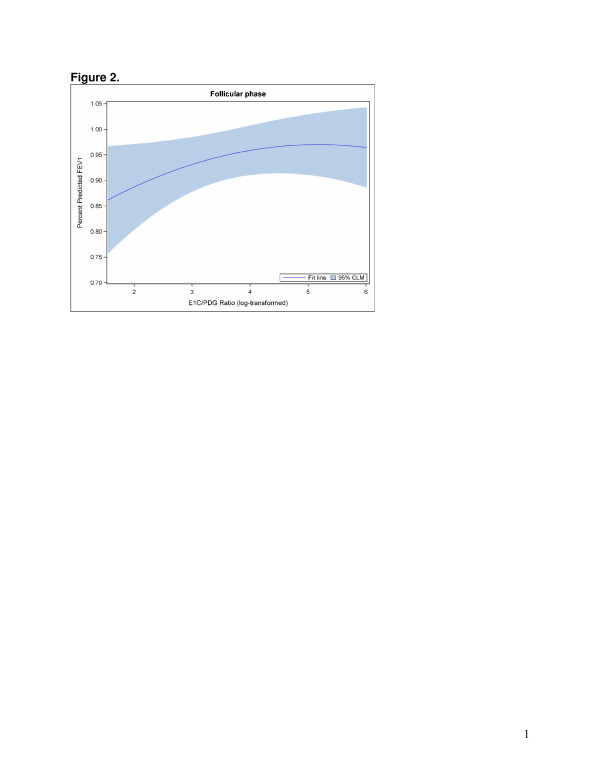
**The log of PDG hormone level (x axis) associated with percent predicted FEV1 (y axis), adjusted for log of E1C in the follicular phase among all women.** For purposes of illustrating the relationship between log PDG and FEV1, menstrual segment day is held constant at 7 days prior to ovulation and log E1C is set to its mean value.

**Figure 4:**
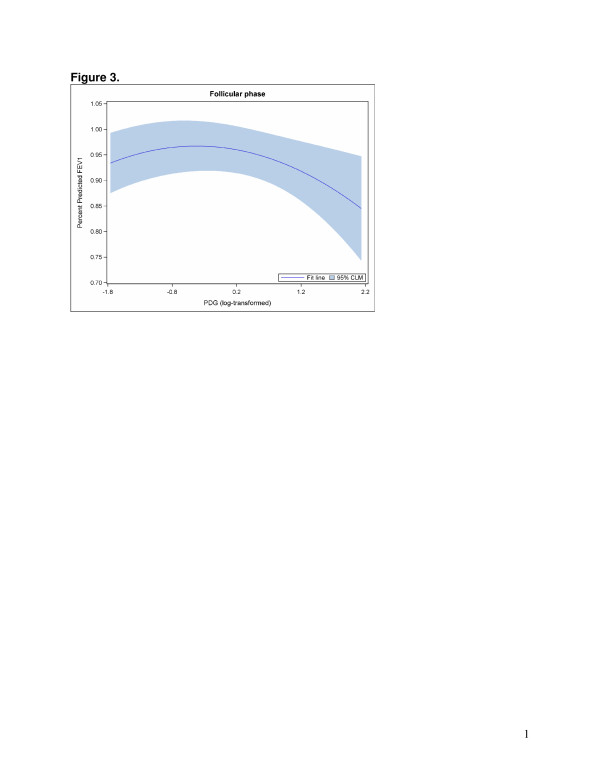
**The log of E1C/PDG ratio of hormone levels (x axis) associated with percent predicted FEV1 (y axis) in the follicular phase among women with no prior asthma diagnosis.** For purposes of illustrating the relationship between log ratio and FEV1, menstrual segment day is held constant at 7 days prior to ovulation.

**Figure 5:**
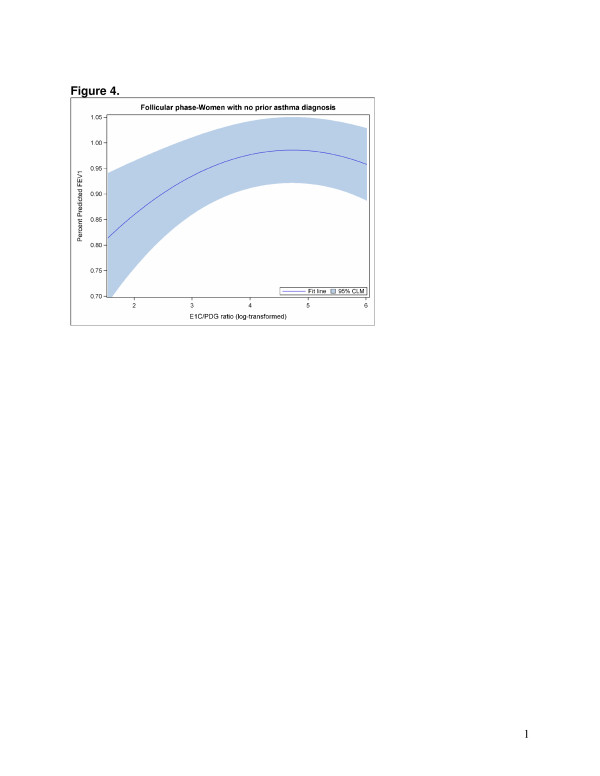
**The log of E1C hormone level (x axis) associated with percent predicted FEV1 (y axis) in the follicular phase among women with no prior asthma diagnosis.** For purposes of illustrating the relationship between log ratio and FEV1, menstrual segment day is held constant at 7 days prior to ovulation.

**Figure 6:**
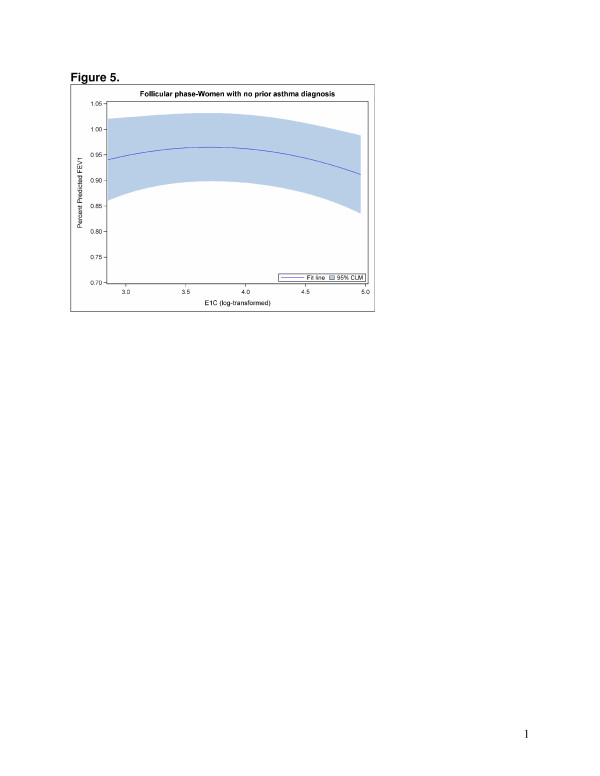
**The log of PDG hormone level (x axis) associated with percent predicted FEV1 (y axis) in the follicular phase among women with no prior asthma diagnosis.** For purposes of illustrating the relationship between log PDG and FEV1, menstrual segment day is held constant at 7 days prior to ovulation.

The GEE models that examined hormone levels were best fit with a quadratic term to accommodate the nonlinear relationships between sex hormones and FEV1. Direct model interpretation is complicated due to the quadratic term. Overall, statistically significant associations were found between the E1C/PDG ratio (Figure 2) and PDG (adjusted for E1C) (Figure 3) and FEV1 in the follicular phase (Table 4). However, different patterns emerged when the models were stratified by asthma status. Among non-asthmatic women, statistically significant associations were found between the E1C/PDG ratio (Figure 4) as well as E1C (Figure 5) and PDG (Figure 6) and FEV1 in the follicular phase (Table 4). Although not statistically significant, only the association with E1C persisted in the luteal phase. Additionally, E1C was associated with FEV1 in the five days prior to bleeding only among non-asthmatic women (Table 5). Among asthmatic women, there were no statistically significant associations between the sex hormones and FEV1 in either phase (Table 4) or in the 5 days prior to bleeding (Table 5).

**Table 4:** **Models of percent predicted FEV1 in relation to hormone measurements from sample collected on the same day as the FEV1 measurement was collected**^**a**^

	Independent variable(s)	Follicular Phase Beta (s.e.)			Luteal Phase Beta (s.e.)		
	**All women**	Term		p	Term		p
1	Ratio (log transformed)	Linear	0.085 (0.043)	0.049	Linear	−0.020 (0.022)	0.37
		Quadratic	−0.008 (0.004)	0.059	Quadratic	0.002 (0.003)	0.49
2	PDG (log transformed)	Linear	−0.001 (0.007)	0.91	Linear	0.008 (0.012)	0.49
		Quadratic	−0.016 (0.005)	0.006	Quadratic	−0.001 (0.004)	0.77
3	E1C (log transformed)	Linear	0.055 (0.120)	0.65	Linear	0.154 (0.130)	0.24
		Quadratic	−0.007 (0.015)	0.65	Quadratic	−0.017 (0.016)	0.28
4	PDG (log transformed)	Linear	−0.016 (0.016)	0.32	Linear	0.006 (0.013)	0.67
		Quadratic	−0.019 (0.006)	0.001	Quadratic	−0.001 (0.004)	0.87
	E1C (log transformed)	Linear	0.122 (0.126)	0.33	Linear	0.109 (0.113)	0.34
		Quadratic	−0.012 (0.015)	0.44	Quadratic	−0.012 (0.014)	0.40
	**No Prior Asthma Diagnosis**						
1	Ratio (log transformed)	Linear	0.161 (0.051)	0.002	Linear	−0.034 (0.037)	0.35
		Quadratic	−0.017 (0.005)	0.001	Quadratic	0.003 (0.006)	0.59
2	PDG (log transformed)	Linear	−0.006 (0.011)	0.55	Linear	0.024 (0.013)	0.07
		Quadratic	−0.024 (0.006)	<0.001	Quadratic	−0.004 (0.005)	0.38
3	E1C (log transformed)	Linear	0.251 (0.130)	0.055	Linear	0.344 (0.189)	0.06
		Quadratic	−0.034 (0.016)	0.036	Quadratic	−0.039 (0.023)	0.08
4	PDG (log transformed)	Linear	−0.021 (0.019)	0.29	Linear	0.019 (0.014)	0.18
		Quadratic	−0.025 (0.006)	<0.001	Quadratic	−0.003 (0.004)	0.49
	E1C (log transformed)	Linear	0.270 (0.094)	0.004	Linear	0.198 (0.160)	0.22
		Quadratic	−0.031 (0.011)	0.006	Quadratic	−0.023 (0.020)	0.24
	**Prior Asthma Diagnosis**						
1	Ratio (log transformed)	Linear	−0.023 (0.029)	0.43	Linear	−0.013 (0.017)	0.43
		Quadratic	0.004 (0.004)	0.36	Quadratic	0.003 (0.003)	0.21
2	PDG (log transformed)	Linear	0.004 (0.012)	0.74	Linear	−0.013 (0.010)	0.19
		Quadratic	−0.006 (0.006)	0.37	Quadratic	0.005 (0.003)	0.17
3	E1C (log transformed)	Linear	−0.092 (0.133)	0.49	Linear	−0.096 (0.097)	0.32
		Quadratic	0.013 (0.016)	0.40	Quadratic	0.014 (0.011)	0.20
4	PDG (log transformed)	Linear	−0.005 (0.019)	0.81	Linear	−0.014 (0.012)	0.25
		Quadratic	−0.008 (0.008)	0.35	Quadratic	0.004 (0.004)	0.30
	E1C (log transformed)	Linear	−0.059 (0.157)	0.71	Linear	0.010 (0.022)	0.64
		Quadratic	0.010 (0.018)	0.58	Quadratic	0.002 (0.003)	0.58

a. Four models are presented for each time period of hormones: 1) Only the ratio (E1C/PDG) is in the model; 2) only PDG is in the model; 3) only E1C is in the model; and 4) both PDG and E1C are in the model.

**Table 5:** **Models of percent predicted FEV1 in relation to hormone measurements from sample collected on the same day as the FEV1 measurement was collected**^**a**^

	Independent variable(s)	5 Days before Onset of Menses		
	**All women**	Beta (s.e.)		
		Term		p
1	Ratio (log transformed)	Linear	0.022 (0.018)	0.22
		Quadratic	−0.001 (0.002)	0.77
2	PDG (log transformed)	Linear	−0.006 (0.011)	0.60
		Quadratic	−0.007 (0.006)	0.22
3	E1C (log transformed)	Linear	0.654 (0.284)	0.021
		Quadratic	−0.081 (0.035)	0.021
4	PDG (log transformed)	Linear	−0.020 (0.009)	0.017
		Quadratic	−0.001 (0.003)	0.80
	E1C (log transformed)	Linear	0.647 (0.268)	0.016
		Quadratic	−0.078 (0.033)	0.017
	**No Prior Asthma Diagnosis**			
1	Ratio (log transformed)	Linear	0.021 (0.041)	0.62
		Quadratic	−0.001 (0.005)	0.84
2	PDG (log transformed)	Linear	−0.017 (0.018)	0.33
		Quadratic	−0.008 (0.008)	0.28
3	E1C (log transformed)	Linear	1.35 (0.229)	<0.001
		Quadratic	−0.159 (0.028)	<0.001
4	PDG (log transformed)	Linear	−0.015 (0.017)	0.37
		Quadratic	0.001 (0.002)	0.65
	E1C (log transformed)	Linear	1.33 (0.221)	<0.001
		Quadratic	−0.157 (0.027)	<0.001
	**Prior Asthma Diagnosis**			
1	Ratio (log transformed)	Linear	0.019 (0.032)	0.56
		Quadratic	−0.007 (0.010)	0.52
2	PDG (log transformed)	Linear	0.030 (0.056)	0.59
		Quadratic	−0.003 (0.011)	0.75
3	E1C (log transformed)	Linear	0.083 (0.417)	0.84
		Quadratic	−0.007 (0.057)	0.90
4	PDG (log transformed)	Linear	−0.013 (0.024)	0.58
		Quadratic	0.001 (0.006)	0.91
	E1C (log transformed)	Linear	0.075 (0.395)	0.85
		Quadratic	−0.004 (0.005)	0.94

a. Four models are presented for each time period of hormones: 1) Only the ratio (E1C/PDG) is in the model; 2) only PDG is in the model; 3) only E1C is in the model; and 4) both PDG and E1C are in the model.

Results were unchanged after excluding samples from three women without complete menstrual segments.

In the open-ended comments made at the end of the study, women reported overall that the study protocol was “easy” or “simple”. Participants commented that urine collection was complicated when travel occurred. Several commented that because urine collection was not “clean catch” it was not a burden. Some women commented that having the pink rubber duck in the bathroom was a good reminder while one woman commented that only having the urine collection cup in the bathroom worked as a reminder.

## Discussion

Detailed evidence of a direct relationship between sex hormones and lung function within-woman over time has been missing from the discussion of the role sex hormones play in asthma incidence and exacerbation. This research establishes methods, including statistical methods and daily hormone measurements, to be applied to future research of this important, yet poorly understood, relationship.

Our results suggest an association between sex hormones and FEV1 that varies by asthma status and menstrual phase (follicular or luteal). Interestingly, there were no statistically significant associations between sex hormones and FEV1 in women who reported a prior doctor diagnosis of asthma; associations were found among women without a prior asthma diagnosis. The differences in associations between sex hormones and FEV1 by asthma status suggests a worthy avenue to pursue in explaining why there may be worsening of asthma around times of hormonal change such as menses and pregnancy. Why might the associations between sex hormones and lung function vary by asthma status? Perhaps our asthmatic women were well controlled and thus might have different relationships between their sex hormones and their FEV1 than uncontrolled asthmatics would have. Further, a larger sample would allow detection of weaker associations and a study with at least 2 consecutive menstrual cycles would be more informative.

Mandhane et al. hypothesized there would be changes in asthma characteristics over the course of a menstrual cycle and that those changes would be “blunted” in women taking oral contraceptives (OCs – all were combined estrogen and progesterone formulations) [13]. They studied 17 asthmatic women (9 took OCs) over the course of a menstrual cycle. Daily measurements of exhaled nitric oxide (eNO) and spirometry (FEV1/FVC ratio) were performed and daily sex hormone measurements were determined from saliva. Among OC users, 17β-estradiol and progesterone levels were not associated with eNO; however, among women not using OCs, an increase in progesterone was associated with an increase in eNO and an increase in 17β-estradiol was associated with a decrease in eNO. No associations were reported between either of the sex hormones and the FEV1/FVC ratio in either OC users or non-users. The authors did not estimate ovulation among those not using OCs and did not consider menstrual “phase” as a potential effect modifier.

Farha et al. collected lung function measurements (spirometry, gas transfer, FeNO) once weekly for four to five weeks from 13 asthmatic and 10 non-asthmatic women – some who were taking hormonal contraceptives [14]. They reported that women with asthma experienced cyclic changes in airflow and gas transfer and suggested that the data supported hormonal effects on lung function. They suggested that the cyclic mechanisms differ between asthmatic and non-asthmatic women. The lack of daily measurements limits the strength of their conclusions.

Our research establishes methods that provide the best data to investigate hormone related asthma effects such as premenstrual worsening of asthma. Most prior research in this area has been limited by poor (or lack of) confirmation of menstrual phase, the lack of actual measurement of sex hormone levels throughout a cycle, use of a measurement of lung function other than percent predicted FEV1, statistical methods that do not take into account repeated measures and the failure to include non-asthmatic women [15-19]. Some of these studies have also focused on comparison of symptoms between asthmatic OC users and non-users generally finding fewer symptoms and better asthma outcomes in OC users [20].

Numerous epidemiologic studies have measured daily hormones to either study fertility or ovarian function [7,8] while prior research on menstrual cycle phase and asthma has used the LMP date to approximate a woman’s menstrual cycle phase. LMP date is used to estimate the date of expected ovulation and thus the phase is established. Although this method is very inexpensive and it is easy to collect data on LMP, there are numerous problems with this approach. Recent data demonstrate that cycle length is not predictive of day of ovulation. In the Early Pregnancy Study, daily urine was collected to measure hormones to study subclinical and early pregnancy loss [7]. In this study, Wilcox et al found that among the 69 cycles that were 28 days long, only 10% of the cycles were associated with ovulation 14 days before the next menses. They also reported that the time from ovulation to the next menses ranged from menstrual cycle days 10 to 22 in these 28 day cycles. Using the same data, Harlow et al. examined the hormonal patterns of 28 cycles with follicular phases of 24 days or more [21]. There were five different hormonal patterns observed among these cycles. Four of the cycles >38 days appeared to be “double cycles” with no bleeding. They further report that the daily estrogen profiles in long cycles are heterogeneous.

These data provide evidence that using LMP date is not a valid method to estimate either hormone levels or menstrual phase in women. The follicular phase can be highly variable – even in ovulatory cycles. Thus, LMP is not a valid measure to estimate menstrual phase and infer hormone patterns. Separately, fertility monitors only indicate the timing of probable ovulation. They also require the use of a daily urine specimen, but do not provide measurements of all important sex hormones or their ratios which would be critical for their study. Fertility monitor accuracy, especially in research studies, has not been well established [22]. Daily hormone measurements are needed if an accurate relationship between actual levels of hormones (and their ratios) and lung function/asthma symptoms are to be determined.

In our study, daily spirometry was not performed by a certified provider due to financial constraints. Women were asked to complete only one useable reading. While these are limitations, women did use the same device for the entire protocol and all received the same instructions to use the device. Also, as part of the study protocol, women met with the research assistant three times per week and the research assistant was able to discuss protocol compliance, as well as confirm proper use of the device. While the device meets American Thoracic Society (ATS) recommendations for accuracy and precision in measuring peak flow, it is not clear if validation testing has been conducted for FEV1. However, we examined within-woman patterns based on the same device used day after day. It would not have been logistically or economically feasible to have women perform spirometry on an ATS approved spirometer daily under the supervision of a NIOSH certified technician. We propose that since we are conducting research and not providing clinical care or medical advice based on results from the device, use of this device is appropriate.

While including only 13 women in our analyses is a limitation, use of daily hormone measurements and repeated FEV1 measurements over a single menstrual segment for each woman are innovative. The use of longitudinal analyses to examine within woman associations provides strong evidence of patterns. We also examined whether absolute levels or ratios of hormone levels were associated with FEV1 levels which is novel.

In summary, our analyses of daily hormone and lung function measurements provide early evidence of differences between asthmatic and non-asthmatic women over menstrual segments. Most importantly, the methods demonstrated here provide optimal evidence for investigating the role of hormones in lung function in asthmatic and non-asthmatic women. These data provide preliminary evidence that the associations between sex hormones and percent predicted FEV1, a measure of lung function, vary by asthma status and phase of the menstrual cycle. The study of contiguous cycles from a larger group of women in their 20s and 30s (most likely to ovulate) would provide the strongest evidence for determining the relationships between sex hormones and lung function, which would lead to insight into the phenomena of so-called hormone-affected asthma.

We learned a series of lessons from this pilot study. First, women should wait until just before their period to start the protocol; however, we should not allow women to start the protocol if they are bleeding as a full menstrual cycle may not be captured during the study. Also, it would be ideal to select times when women do not plan to be out of town during participation. If they must travel, plans should be developed with the participant to collect and store their urine during travel. Necessary supplies, such as ice packs and coolers, should be provided for travel collection and storage. The study protocol should be extended to at least 2 full menstrual cycles (capture 3 bleeds) to allow analyses of within-woman cycle-to-cycle variability of associations.

Sample size calculations, for which our data should prove helpful, should take into consideration that some cycles will be anovulatory or will not permit estimation of ovulation day (18.8% in our study). For the FEV1 measurements, women should perform the exhale function 3 times to allow the investigator to select the best measurement from the electronically stored data. Finally, baseline spirometry with Albuterol challenge would also be useful in describing the lung function of the population and the severity of asthmatics included in the study.

## Abbreviations

E1C: Estrone; PDG: Pregnanediol-glucuronide; FEV1: Forced Expiratory Volume at 1 second.

## Competing interests

The authors declare they have no competing interests.

This work was funded by the Feldstein Medical Foundation and the Fund for Henry Ford Hospital. Neither was involved in any aspect of the study.

## Authors’ contributions

GW designed the study and drafted the manuscript; EH collected all data and made recommendations to the protocol; HB, provided clinical expertise in data interpretation and in drafting the manuscript; CCJ, provided consultation on design and manuscript preparation; RS and EZ provided clinical expertise in data interpretation and in drafting the manuscript; and SH performed all analyses. All authors read and approved the final manuscript.
